# Personalized Medicine in ANCA-Associated Vasculitis ANCA Specificity as the Guide?

**DOI:** 10.3389/fimmu.2019.02855

**Published:** 2019-12-06

**Authors:** Zachary S. Wallace, John H. Stone

**Affiliations:** ^1^Clinical Epidemiology Program, Mongan Institute, Boston, MA, United States; ^2^Rheumatology Unit, Division of Rheumatology, Allergy, and Immunology, Boston, MA, United States; ^3^Massachusetts General Hospital, Boston, MA, United States; ^4^Harvard Medical School, Boston, MA, United States

**Keywords:** ANCA–associated vasculitis, vasculilis, personalized medicine, genetics, pathogenesis

## Abstract

Anti-neutrophil cytoplasmic antibody (ANCA)-associated vasculitis (AAV) is a small- to medium-vessel necrotizing vasculitis responsible for excess morbidity and mortality (1). The AAVs, which include granulomatosis with polyangiitis (GPA), microscopic polyangiitis (MPA), and eosinophilic granulomatosis with polyangiitis (EGPA), are among the most difficult types of vasculitis to treat. Although clinicopathologic disease definitions have been used traditionally to categorize patients into one of these three diagnoses, more recently ANCA specificity for either proteinase 3 (PR3) or myeloperoxidase (MPO) has been advocated for the purpose of disease classification (2). This is because differences in genetics, pathogenesis, risk factors, treatment responses, and outcomes align more closely with PR3- or MPO-ANCA type than with the clinocopathologic diagnosis. Moreover, classifying patients as GPA or MPA can be challenging because biopsies are not obtained routinely in most cases and existing classification systems can provide discrepant classification for the same patient (3). In this review, we address the recent literature supporting the use of ANCA specificity to study and personalize the care of AAV patients (Table 1). We focus particularly on patients with GPA or MPA.

## Genetic Differences Between PR3- and MPO-ANCA+ Patients

An estimated 20% of AAV risk is due to genetic factors (4). Several genome-wide association studies (GWAS) have identified functional genetic variants leading to altered gene expression or protein function that are thought to be relevant to AAV pathogenesis, presumably explaining the association between these variants and AAV risk (4–6). Across these studies, AAV risk has been most strongly associated with gene variants in the MHC class II region, but non-MHC associations have also been identified. The preponderance of evidence suggests that ANCA type distinguishes two groups characterized by unique genetics better than clinical phenotype (4–6).

**Table 1:** Distinguishing features between PR3-ANCA+ and MPO-ANCA+ AAV.

**Feature**	**PR3-ANCA (associated with C-ANCA pattern)**	**MPO-ANCA (associated with P-ANCA pattern)**
Age at Diagnosis	• 45–55 years	• 60–65 years
Racial/Geographic Differences	• ↑ Northern Europe and Americas	• ↑ Asia and Southern Europe
Genetic Differences	• *HLA-DPA1* and *DPB1* • *SERPINA1* • *PRTN3*	• *HLA-DQA2* and *DQB1*
Pathogenesis	• Normally expressed on PMN cell surface • Granulomatous inflammation common • ↑ IL-6, IL-15, IL-18, IL-18 binding protein, sIL-2 receptor a, gCSF, and mCSF	• Not normally expressed on PMN cell surface • Fibrotic manifestations observed (e.g., ILD) • ↑ sIL-6 receptor, sTNF-receptor type II, neutrophil gelatinase–associated lipocalin, and soluble intercellular adhesion molecule
Risk Factors	• ± *S. aureus*	• Drugs (e.g., hydralazine, levamisole, propylthiouracil)
Clinical Phenotype	• >> GPA (~90%)	• >>> MPA (~100%)
Organ Involvement	• Ear, nose, and sinus disease • Upper airway involvement • Pulmonary nodules/cavitary lesions	• Pulmonary fibrosis • Bronchiectasis • Renal disease (↑ severity and chronicity)
Response to RTX or CYC	• RTX > CYC for remission induction	• RTX = CYC for remission induction
Outcomes	• ↑ Relapse and treatment failure • Rising titer may predict flare if RTX-treated	• ↑ Non-fatal CVD events • ↑ Death to CVD

A recent study confirmed previous reports that PR3-ANCA+ but not MPO-ANCA+ disease is associated with gene variants in *HLA-DPA1* and *DPB1*. A tri-allelic *HLA-DPB1* haplotype explained much of the genetic risk in patients with AAV. In contrast, MPO-ANCA+ disease is associated with *HLA-DQA2* and *DQB1* variants (4, 5). Non-MHC variants such as those in the *SERPINA1* and *PRTN3* genes have been associated with PR3-ANCA+ but not MPO-ANCA+ disease, but variants in *PTPN22* are observed in both MPO- and PR3-ANCA+ disease (4, 5). Functional studies have expanded upon previous GWAS studies and confirmed the potential pathogenic link between genetic variants and AAV (6).

Given the associations between genetic variants and ANCA specificity, genetic testing may play a future role in identifying patients at risk for AAV. In fact, the presence of several of these variants (e.g., MHC and non-MHC) in the same individual increases the odds that the individual will develop AAV (4). However, additional studies are necessary to understand how genetic testing might be used in the clinical setting. Moreover, our knowledge of genetic associations in AAV stems from studies of patients of European descent and may be difficult to extrapolate to patients with other ancestry. One previous case-control study found that genetic variants at *DRB1* might predispose African American patients to PR3-ANCA+ AAV (7), but additional studies in patients of non-European descent are needed.

## Pathogenesis of PR3- and MPO-ANCA+ AAV

The pathogenesis of AAV is complex and the precise cause or causes remain unknown, but MPO- and PR3-ANCA are generally considered to have substantial roles in the pathophysiology of most patients' disease (8). Direct proof of a relationship between the presence of these antibodies and the initiation of disease in humans, however, remains lacking, despite the fact that compelling animal models for AAV exist. This is particularly true for MPO-ANCA, as discussed below (9).

MPO- and PR3-ANCA+ AAV appear to share many features of pathogenesis, yet certain differences have also been observed. Myeloperoxidase and proteinase 3, the targets of MPO- and PR3-ANCA, respectively, are both found in neutrophil granules and monocyte lysosomes. PR3 is normally expressed on the neutrophil cell surface, more so in PR3-ANCA+ patients than healthy controls. In contrast, MPO is not spontaneously expressed on neutrophil cell surfaces but surface MPO expression is detectable after neutrophil activation (10).

In AAV, the binding of MPO- or PR3-ANCA to neutrophils induces activation and degranulation as well as adhesion and transmigration of neutrophils across the vascular endothelium, culminating in endothelial cell damage. The role of monocytes in AAV is less well understood. The pathogenic importance of MPO-ANCA is supported by the ability of these antibodies to induce a vasculitis syndrome resembling AAV when MPO-ANCA are transferred into experimental mouse models (9). The development of a similar animal model for PR3-ANCA+ AAV has been elusive to date, in part due to differences in PR3 expression in mice and humans.

Several additional observations support the importance of PR3- and MPO-ANCA in the pathogenesis of AAV. These include: (1) the great majority of patients with AAV are MPO- or PR3-ANCA+ (2, 11) there are consistent differences in clinical features of AAV according to ANCA type (see below); (3) B-cell targeted therapies and/or plasma exchange are efficacious in both PR3- and MPO-ANCA+ AAV (4, 12, 13) there is some correlation between ANCA titer and disease activity (see below); (5) transplacental transfer of MPO-ANCA is reported to have caused AAV in a newborn (6, 14); PR3-ANCA+ antibodies are known to appear in patients' blood years before clinical presentation (15); and (7) genetic variants in proteinase 3, the antigenic target of PR3-ANCA, are associated with PR3-ANCA+ AAV (see above). However, the presence of MPO- or PR3-ANCA positivity does not always correlate with disease activity, suggesting that multiple factors are necessary to induce vasculitic and granulomatous features of AAV. Such factors include genes, infections, medications, environmental exposures, the epitope specificity of ANCA, and almost certainly others (8).

Neutrophil extracellular traps (NETs) are increasingly recognized as important for the pathogenesis of autoimmune conditions, including both MPO- and PR3-ANCA+ AAV (16, 17). In normal individuals, NETs are immunogenic and have a role in trapping and killing invading extracellular microbes. Notably, NETs can activate certain immune cells, including autoreactive B cells (16, 17), and cause end-organ damage. Spontaneous NET formation is observed more often in AAV patients than in healthy controls, likely because of stimulation of neutrophils by ANCA (16), and correlates with disease activity (17). Upon stimulation, NETs containing PR3 and MPO (16) are released in both the circulation as well as in damaged tissues.

Complement has traditionally not been thought to play a role in the pathogenesis of these “pauci-immune” vasculitides. Neither immunoglobulins nor complement components are observed prominently in the biopsy specimens from patients with AAV. The lack of immunoglobulin and complement in the renal lesions of AAV, for example, contrasts strikingly with the glomerular lesions observed in systemic lupus erythematosus, for example. However, mounting evidence suggests that activation of the alternative pathway is important to the pathogenesis of MPO-ANCA+ and, more recently, PR3-ANCA+ AAV (18, 19). A recent study by Wu et al. suggested that the classical or lectin complement pathways are activated in PR3-ANCA+ but not MPO-ANCA+ AAV (18). Moreover, avacopan, a C5a receptor inhibitor, was found in early phase trials to have efficacy in AAV and have a potential role as a glucocorticoid-sparing drug in remission induction (20). The results of an ongoing phase 3 randomized controlled trial evaluating its efficacy for remission induction will be an important proof-of-concept advance in our understanding of the role of complement activation in AAV (21).

Cytokine profiles may highlight potential differences in pathogenesis between MPO- and PR3-ANCA+ patients. Berti et al. recently compared differences in serum cytokine profiles associated with inflammation, proliferation, vascular injury, and tissue damage and repair among AAV patients grouped according to ANCA type or clinical diagnosis (22). Differences according to phenotype (e.g., PR3- vs. MPO-ANCA+ and GPA vs. MPA) were observed regardless of whether ANCA type or clinicopathologic condition was used to group patients, but the differences were more striking when PR3- and MPO-ANCA patients were compared to one another.

In the study by Berti et al., nine biomarkers were higher among the PR3-ANCA+ subset (22). These included interleukin (IL)-6, granulocyte–macrophage colony-stimulating factor, IL-15, IL-18, CXCL8/IL-8, CCL17/thymus and activation–regulated chemokine, IL-18 binding protein, soluble IL-2 receptor a, and nerve growth factor b. Four cytokines were higher in the MPO-ANCA+ subset, including soluble IL-6 receptor, soluble tumor necrosis factor receptor type II, neutrophil gelatinase–associated lipocalin, and soluble intercellular adhesion molecule. In multivariate-adjusted analyses, no cytokine levels remained significantly associated with either GPA or MPA, but several associations between cytokines and ANCA-type persisted. Additional studies are necessary to further validate these observations, particularly in larger MPO-ANCA+ cohorts.

In conclusion, the current pathogenic model of AAV suggest that MPO- and PR3-ANCA+ vasculitis share many similar pathogenic features. However, recent studies suggest that there may also be differences in complement activation and cytokine profiles according to ANCA type. Additional studies are necessary to clarify how pathogenesis may differ according to ANCA type. Differences in pathogenesis between PR3- and MPO-ANCA+ patients may identify novel treatments guided by ANCA specificity.

## AAV Risk Factors

Several potential risk factors have been associated with the development of AAV, including environmental, drug, and infectious exposures.

### Silica

Silica exposure, typically related to occupational history, has been associated with AAV in several studies. Indeed, a recent meta-analysis found that silica exposure was associated with a 2.6-fold higher odds (OR 2.6, 95% CI: 1.5–4.4) of AAV (23). This observation was true for MPA and GPA patients, suggesting that similar risk exists for both MPO- and PR3-ANCA+ subjects. In another study, MPO-ANCA+ disease was more common than PR3-ANCA+ disease (24) among cases with high silica exposure, but additional studies of this question would be useful.

### Staphylococcus aureus

There is a long-standing interest in understanding potential associations between microbes, particularly chronic nasal carriage of *Staphylococcus aureus*, and the risk of AAV and flare. These suspected associations date back to early observations of infectious symptoms and secondary sinonasal infections in GPA patients with sinonasal disease (25). Subsequently, a small clinical trial in GPA, the majority of whom were presumably PR3-ANCA+, found that trimethoprim/sulfamethoxazole was associated with a 70% (HR 0.3, 95% CI: 0.1–0.8) reduction in risk of flare compared to placebo. These findings have been interpreted as support of the hypothesized role of *S. aureus* or other microbes as risk factors for AAV relapse (26). However, it has been noted that the effects of trimethoprim/sulfamethoxazole on disease activity might be mediated through mechanisms other than reducing *S. aureus* carriage, given that changes in *S. aureus* carriage on antibiotics did not necessarily relate to subsequent flare.

More recently, in a sub-study of two randomized clinical trials, GPA patients with chronic nasal *S. aureus* carriage were observed to have a higher risk of relapse than GPA patients without chronic *S. aureus* carriage (27). Again, these findings suggest an association between chronic *S. aureus* carriage and relapse risk, but the authors propose that that an underlying genetic confounder might be responsible for this observation. In GPA, and therefore likely PR3-ANCA+ AAV, we can therefore only surmise that chronic nasal carriage of *S. aureus* may be associated with the risk of flare, but further studies are needed to account for potential confounders of this observed association. There is no strong evidence base to suggest that *S. aureus* or other infections, however, are risk factors for GPA or AAV generally.

### Medication-Induced AAV

A number of drug exposures, including prescribed medications and illicit substances, have been associated with AAV, though well-designed studies assessing the association between these exposures and risk of AAV are lacking. Case series and anecdotal experience strongly suggest potential associations between drug exposures, particularly hydralazine (28), propylthiouracil (28, 29), and levamisole (typically when in adulterated cocaine) (30). The link between these medications and AAV appears to be far stronger for MPO-ANCA+ AAV than for PR3-ANCA+ AAV. Extremely high titers of MPO are often reported in these cases. In one single-center study, 13 of 30 (43%) patients with the highest MPO-ANCA titers in a large hospital's ANCA lab had been exposed to hydralazine or propylthiouracil (28).

Levamisole-contaminated cocaine has also been associated with AAV. This drug-induced syndrome is manifested often by large-joint arthralgias and cutaneous lesions, purpuric earlobe lesions, and frequently MPO-ANCA positivity but often dual positivity (50% were PR3- and MPO-ANCA+ in one study) (30). The presence of both MPO- and PR3-ANCA positivity is not seen in all cases of drug-induced AAV, but dual-positivity should raise suspicion for a drug culprit. It is important to note that the presence of ANCA positivity in the setting of drug exposure can occur without clinical features of vasculitis and is not diagnostic of AAV. The MPO-ANCA in propylthiouracil therapy, for instance, may have features that distinguish it from the pathogenic MPO-ANCA seen in classic AAV (29).

In summary, several risk factors for AAV have been proposed and these may differ according to ANCA type (e.g., *S. aureus* in PR3-ANCA+, drugs in MPO-ANCA+). However, environmental exposures, particularly to silica, appear to be a common risk factor in both PR3- and MPO-ANCA+ AAV. Additional well-designed studies are needed to better characterize environmental, infectious, and other exposure-related risk factors in AAV, particularly according to ANCA type.

## ANCA Testing for the Diagnosis and Monitoring of AAV

The initial discovery of ANCA among patients with clinical syndromes that would be characterized as GPA or MPA was a major milestone in the diagnosis and management of these conditions (31). Following the discovery of ANCA and spreading availability of testing, the diagnosis of GPA or MPA was increasingly made with confidence in the proper clinical setting, often without a biopsy.

The classic approach to ANCA testing is a two-step process (32). First, indirect immunofluorescence (IIF) is performed to detect a cytoplasmic or peri-nuclear ANCA pattern. Second, immunoassays of samples positive for ANCA by IIF are performed to confirm the IIF results and to detect ANCA specificity (e.g., PR3-ANCA or MPO-ANCA). However, accumulating evidence suggests that the test performance (e.g., receiver operating characteristic curves) of contemporary immunoassays is quite strong and less susceptible to inter-reader variability and other potential sources of imprecision than IIF (33). For instance, in a study by Damoiseaux et al., the area under the curve (AUC) of immunoassays for PR3- or MPO-ANCA was between 94 and 96%, whereas the AUC for IIF was between 84 and 92% (33). A two-step process for ANCA testing has not been found to improve test performance (33, 34). Therefore, a one-step process using only immunoassay testing for PR3- or MPO-ANCA without IIF is sufficient for diagnosing AAV. In addition to test performance, it is also important to consider the test results appropriately. Though PR3- and MPO-ANCA test results are often interpreted as positive or negative, the test performance may vary according to titer such that increasing titers may more accurately classify patients according to the correct diagnosis (34).

The role of serial ANCA testing in the management, as opposed to diagnosis, of AAV patients remains poorly defined and controversial. In a *post-hoc* analysis of the Wegener Granulomatosis Etanercept Trial (WGET) trial in which patients with GPA were randomized to conventional therapy (cyclophosphamide or methotrexate) or conventional therapy plus etanercept (35), PR3-ANCA titers correlated with disease activity and both PR3- and MPO-ANCA titers decreased during remission induction (36). Notably, the vast majority (~73%) of patients in WGET were PR3-ANCA+ (35). A meta-analysis that includes *post-hoc* analyses of WGET as well as other studies found that a rise in ANCA levels in patients in remission was associated with a positive likelihood ratio of 2.8 (95% CI: 1.7–4.9) of a future relapse; the absence of a rise in ANCA was associated a negative likelihood ratio of 0.5 (95% CI: 0.3–0.9) of having a future relapse (37). Becoming ANCA negative, and even staying ANCA negative during follow-up, has not been observed to be a reliable indicator that a patient will achieve or maintain remission (36, 37).

The utility of repeat testing may differ according to ANCA type, especially with contemporary treatment strategies. Findings from the Rituximab in ANCA-Associated Vasculitis (RAVE) trial provided additional insights into the potential value of serial ANCA testing. In the RAVE trial, MPO- and PR3-ANCA+ patients were randomized to remission induction with either rituximab (RTX) or cyclophosphamide followed by azathioprine (CYC/AZA) (12). Approximately 67% of patients were PR3-ANCA+ in RAVE. Similar to observations from WGET, RAVE patients who became ANCA negative were not more likely to achieve clinical remission at 6 months (12). However, differences in the likelihood of becoming ANCA negative were observed according to ANCA type and treatment. In particular, PR3-ANCA+ patients treated with RTX were more likely than those treated with CYC/AZA to become ANCA negative. There was no difference in the rate of becoming ANCA negative among MPO-ANCA+ patients treated with RTX or CYC/AZA (12).

Among PR3-ANCA+ patients treated with RTX in RAVE, a *post-hoc* analysis found that a rise (defined as a doubling) in the PR3-ANCA titer was associated with a higher risk of severe relapse within 1 year, especially in those with a history of renal involvement or alveolar hemorrhage (38). This was not observed among PR3-ANCA+ patients treated with CYC/AZA in RAVE and was not observed in a *post-hoc* analysis of WGET where most patients were PR3-ANCA+ and received CYC for severe disease (36). Thus, the potential utility of serial PR3-ANCA testing may be specific to patients treated with rituximab, as opposed to other therapies.

In summary, the significance of an isolated increase in an ANCA titer without an associated change in symptoms or findings otherwise suggestive of a disease flare is of unclear significance. Certainly not all patients who experience an increase in their ANCA titers will go on to have a disease flare and, if they do, the timing of a flare could be many months to even more than a year following the ANCA titer rise. Therefore, one must weigh the risks and benefits of treatment decisions guided by only ANCA titers (36). The ANCA type and treatment exposure may influence the predictive ability of changes in titers so the utility of serial ANCA measurements may evolve over time as our treatment regimens change. It is important to note that most studies to date evaluating the predictive value of changes in ANCA titers have been limited because of frequency of titer measurements, variations in outcome definition, and the inclusion of mostly PR3-ANCA+ patients.

## Clinical Features

### Demographics

MPO-ANCA+ patients are more likely to be female and, on average, 10 years older than PR3-ANCA+ patients at presentation (39). There are also differences in the distribution of ANCA type according to race and geography such that Japanese, Chinese, and Southern European AAV patients are more likely to be MPO- rather than PR3-ANCA+ when compared with non-Japanese, non-Chinese, and Northern European AAV patients (40). In a population-based study comparing AAV incidence and features in defined geographic regions of the UK and Japan, more than 80% of cases in Japan were MPO-ANCA+. In contrast, more than 66% of cases in the UK were PR3-ANCA+ (40).

### Clinical Phenotype

With regard to clinical phenotype, those who are PR3-ANCA+ more often have a presentation consistent with GPA whereas those who are MPO-ANCA+ tend to have features of MPA. However, ~10% of patients with GPA are MPO-ANCA+; PR3-ANCA+ MPA seems to be a rarer phenomenon (41, 42).

In contrast to MPO-ANCA+ patients, those who are PR3-ANCA+ are more likely to have involvement of ears, nose, sinuses, and throat (3, 39, 43). Whereas both MPO- and PR3-ANCA+ patients can have lung involvement, those who are MPO-ANCA+ more often present with features of interstitial lung disease (e.g., fibrosing lung disease) rather than cavitary lesions and/or nodules characteristic of PR3-ANCA+ disease (44, 45). Evolving literature suggests that MPO-ANCA+ patients are at higher risk for bronchiectasis, which is often present prior to AAV presentation. In two recent cohort studies, MPO-ANCA+ subjects were found to have bronchiectasis more often than PR3-ANCA+ subjects (44, 46). In one, only MPO-ANCA+ subjects had bronchiectasis (46). In the other, MPO-ANCA+ subjects were twice as likely to have bronchiectasis (31% vs. 15%) and the bronchiectasis was more severe among the MPO-ANCA+ subjects (44). The high proportion of MPO-ANCA+ patients with bronchiectasis raises the question of whether it might predispose to MPO-ANCA+ AAV, be more likely to complicate MPO-ANCA+ AAV, or go undetected for some time before AAV comes to medical attention.

In addition to differences in respiratory tract involvement, MPO-ANCA+ patients more often have renal involvement than PR3-ANCA+ patients. Moreover, among MPO- and PR3-ANCA+ patients with renal involvement, those who are MPO-ANCA+ often present with more severe renal disease, characterized by a lower glomerular filtration rate, greater need for renal replacement therapy (31% vs. 20%), and more chronic appearing lesions on renal biopsy (47). However, ANCA type does not consistently predict the risk of end-stage renal disease (3).

### Features Among Patients With Discordant ANCA Types and Clinical Phenotypes

Though ANCA type is increasingly recognized as a clinically-meaningful and standardized approach to characterizing AAV patients, the combination of ANCA type with clinical phenotype (e.g., GPA or MPA) may identify additional subtypes with unique features (Table 2). Several studies have suggested that there may be differences between MPO-ANCA+ GPA patients compared with those who are PR3-ANCA+ or those who are MPO-ANCA+ and have presentations consistent with MPA (45).

**Table 2:** Potential differences between PR3-ANCA+ GPA and MPO-ANCA+ GPA.

**Feature**	**PR3-ANCA+ GPA**	**MPO-ANCA+ GPA**
Manifestations	• Renal disease more common	• Limited disease more common • Subglottic stenosis more common
Flares	• Higher flare rate	• Lower flare rate

In one single-center cohort study by Schirmer et al., MPO-ANCA+ GPA patients were found to have limited disease more often, to have higher rates of subglottic stenosis, and to have lower rates of renal involvement compared with PR3-ANCA+ GPA patients (45). In a nephrology clinic-based cohort study by Chang et al., Chinese patients with MPO-ANCA+ GPA were found to have less severe renal disease than PR3-ANCA+ GPA patients and a lower risk of progressive renal failure (42). In contrast, disease manifestations did not differ between MPO-ANCA+ and PR3-ANCA+ GPA patients who had been enrolled in two large clinical trials (41) and studied in a *post-hoc* analysis by Miloslavsky et al. These conflicting results with regard to disease manifestations may be related to differences in study design (clinical trial vs. single center cohort study) (48), classification of GPA and MPA, and enrollment criteria. They may also reflect the limitations of attempting to address these questions in studies of small sample sizes.

Discordant associations between ANCA type and clinical phenotype may also have implications for relapse rates. In the study by Miloslavsky et al., MPO-ANCA+ GPA patients flared more often than MPO-ANCA+ MPA patients (41). Due to statistical limitations, this question could not be addressed in the study by Schirmer et al. (45). In the study by Chang et al., MPO-ANCA+ GPA patients had a lower flare rate than PR3-ANCA+ GPA (42).

In summary, reliable interpretations of the results of these small studies that often provide disparate results is difficult. Nevertheless, it is important to note that MPO-ANCA+ GPA patients may have a unique natural history, especially when compared with PR3-ANCA+ GPA patients.

## Response to Treatment According to ANCA Type

The Rituximab in ANCA-Associated Vasculitis (RAVE) trial randomized patients with severe PR3- or MPO-ANCA+ AAV to either rituximab (RTX) or cyclophosphamide/azathioprine (CYC/AZA) for induction therapy. RTX was found to be non-inferior to CYC/AZA for remission induction. In a *post-hoc* analysis of the RAVE trial, however, PR3-ANCA+ patients treated with RTX had a 2-fold higher odds (OR 2.1, 95% CI: 1.0–4.3) of achieving remission at 6 months than those treated with CYC/AZA (39). This was also true among those PR3-ANCA+ patients who were randomized in the setting of relapsing disease. There was no difference between the efficacy of RTX or CYC/AZA among MPO-ANCA+ patients with regard to achieving remission.

There may also be a difference in the efficacy of mycophenolate mofetil for remission induction in MPO-ANCA+ AAV compared with PR3-ANCA+ AAV patients without life-threatening disease (49). In the recent open-label, non-inferiority MYCYC trial, patients were randomized to mycophenolate mofetil or cyclophosphamide for remission induction. Both arms received azathioprine for maintenance therapy after remission induction. Remission rates at 6 months were similar in the mycophenolate mofetil and cyclophosphamide groups (67% vs. 61%) such that the two were found to be non-inferior to one another. Following remission, more patients in the mycophenolate mofetil group relapsed when compared with those in the cyclophosphamide group (33% vs. 19%). This difference, however, was strongly driven by relapses in PR3-ANCA+ patients, 48% of whom relapsed following mycophenolate mofetil compared with 24% following cyclophosphamide. Therefore, it may be that mycophenolate mofetil is a reasonable option for remission induction in patients who are MPO-ANCA+ but may not be ideal for patients who are PR3-ANCA+.

PR3-ANCA+ patients have been found in multiple studies to relapse more often than MPO-ANCA+ patients following remission induction (3, 45, 50). For instance, in one large United States community-based cohort, PR3-ANCA+ patients have been consistently found to have a nearly 2-fold higher risk of relapse than MPO-ANCA+ patients (3, 51). Though this cohort is largely composed of patients with renal involvement, similar observations regarding differences in the risk of relapse between PR3-ANCA+ and MPO-ANCA+ patients have been made in the RAVE trial (12); a cohort composed of patients from several large European clinical trials (52); as well as a recently described large multi-center Spanish cohort (53). All of those studies included patients with both renal and non-renal manifestations.

Patients with PR3-ANCA+ disease may also be more likely to have treatment-refractory disease. The term “treatment-refractory” is often challenging to define and differing definitions have been used across studies. In the RAVE trial, however, the term “early treatment failure” was used to describe patients whose disease was not responding to therapy at the 1 month time point. Eleven of the 12 early treatment failures in the RAVE trial were PR3-ANCA+ (54). Patients with PR3-ANCA+ disease in the RAVE trial also had a 29% chance of failing the primary outcome at 6 months because of the recurrence of active disease (54).

These observations suggest that different treatment approaches may be indicated for patients depending on ANCA type. PR3-ANCA+ patients, in contrast to MPO-ANCA+ patients, may benefit from rituximab rather than cyclophosphamide for remission induction and may also benefit from continued immunosuppression following remission given their increased risk of relapse. It may be reasonable, for example, to consider an extra one-gram infusion of rituximab at 4 months of treatment in the interest of inducing a solid disease remission. Flare rates, however, vary significantly depending on the regimen used to maintain remission.

In the recent MAINRITSAN trials comparing different contemporary maintenance strategies, those using rituximab at fixed doses had relatively low flare rates (3% at 22 months and 10% at 28 months) (55, 56) compared with the approximate 32% rate of relapse at 18 months without maintenance therapy (50) and 29% relapse rate at 28 months with azathioprine as maintenance (55). The vast majority of patients enrolled in these trials were PR3-ANCA+ so it is difficult to assess how flare rates may vary between PR3- and MPO-ANCA+ patients using contemporary maintenance strategies. One single-center experience using continuous B cell depletion with rituximab in MPO- and PR3-ANCA+ AAV patients reported a relapse rate of 20% but the duration of follow-up in this study is not reported (43), nor is relapse rate according to ANCA type. Additional studies are necessary to determine flare rates according to ANCA type using contemporary maintenance strategies and to understand the optimal long-term management of AAV according to ANCA type.

## Long-Term Outcomes According to ANCA Type

As short-term AAV outcomes are optimized, increasing attention has shifted toward improving long-term outcomes. Particular focus has been paid to reducing the incidence of end-stage renal disease (ESRD) and death in AAV.

Over the last two decades, renal survival in AAV has improved, such that fewer patients are developing ESRD (57). As mentioned, MPO-ANCA+ patients with biopsy-proven disease typically have more chronic, as opposed to active, renal lesions at the time of diagnosis (47, 58–60) when compared to PR3-ANCA+ patients. However, in a large cohort study by Rhee et al., there was no difference in renal survival when MPO- and PR3-ANCA+ patients were compared both in unadjusted and adjusted analyses (aHR 0.92, 95% CI: 0.6–1.5) (57). In that study, the most important predictor of long-term renal survival was renal function at presentation. Similar observations have been made in other studies of ESRD outcomes associated with AAV (61).

Overall, mortality among patients with AAV is approximately 3-fold higher than that of the general population (62) but the gap in survival has improved over the last two decades (63, 64). Both PR3- and MPO-ANCA+ AAV patients are at similarly increased risk of death compared to the general population (47, 65). In other words, PR3- and MPO-ANCA+ AAV patients have a similar risk of death after accounting for differences in age- and sex- distributions between the subgroups (3). However, a recent study suggested that there may be differences in cause-specific death according to ANCA type. While more studies are needed, MPO-ANCA+ patients may be at higher risk for death due to cardiovascular disease even after accounting for differences in renal involvement, age, and sex (65). This observation is also consistent with the results of a prior study which found that MPO-ANCA+ patients may be at higher risk of non-fatal CVD events (66).

Collectively, these findings suggest that to further improve long-term survival in AAV, PR3-, and MPO-ANCA+ patients may benefit from different targeted interventions. Additional studies are necessary to determine whether the management of CVD risk should differ according to ANCA type.

## ANCA-Negative AAV

While ANCA type is increasingly used to classify patients with AAV, it is important to note that a portion of patients with AAV are ANCA negative because the diagnosis of AAV remains based on clinicopathologic features rather than a positive ANCA test. This is especially true in patients with limited AAV and/or non-renal AAV (67). Rates of ANCA negativity in AAV are difficult to estimate because ANCA positivity is often used in AAV diagnostic algorithms. However, ~20% of patients with AAV are thought to be ANCA negative; rates may be as high as 40% in those with limited AAV in historic studies (33, 67, 68). It is important to note that there are an increasing number of methods that can be used to detect PR3- or MPO-ANCA positivity and that the diagnostic test performance characteristics of these methods can vary (33). Therefore, in the setting of high diagnostic suspicion but negative ANCA testing, it may be useful to test for ANCA positivity using an alternative method for ANCA detection (68). There is limited data on the comparison of patients with ANCA negative AAV vs. PR3-ANCA+ AAV vs. MPO-ANCA+ AAV (41). Moreover, many contemporary AAV trials exclude patients who have no history ANCA positivity. Studies of ANCA negative AAV are an important avenue of future investigation.

## Conclusions

ANCA testing is a useful test to establish a diagnosis of AAV in the appropriate clinical setting. ANCA testing also provides important insights into differences in genetic risk, pathogenesis, and response to treatment between PR3- and MPO-ANCA positive patients. A growing body of evidence supports the hypothesis that PR3- and MPO-ANCA+ AAV might represent distinct diseases rather than a single spectrum of disease. A number of research questions can be addressed to further advance our understanding of the potential use of ANCA type for guiding AAV care (Table 3). The available evidence suggests that AAV treatment might be optimized using a personalized approach guided by a patient's ANCA type.

**Table 3:** Future direction of research regarding ANCA type in AAV.

**Topic**	**Research Question**
Genetics and Pathogenesis	• Can unique genetic risk factors be used to identify patients at risk of MPO- or PR3-ANCA+ AAV before the onset of clinical symptoms or findings?
Risk Factors	• Are there modifiable risk factors for AAV that differ according to ANCA type? • How do certain drugs induce an MPO-ANCA+ AAV?
Organ Involvement	• Why might fibrotic lung disease and bronchiectasis be more common in MPO-ANCA+ AAV? • Why is renal disease more severe at presentation in MPO-ANCA+ AAV? • Why does PR3-ANCA+ AAV tend to affect the upper airway more often?
Remission Induction	• Should remission induction treatment decisions be influenced by ANCA type? • Should clinical trials be powered to detect significant differences in treatment arms stratified by ANCA type?
Remission Maintenance	• Should maintenance strategies (continuous vs. tailored immunosuppression) be driven by ANCA type? • Does monitoring PR3- or MPO-ANCA+ titer predict flares depending on treatment strategy used?
Long-Term Outcomes	• Are MPO-ANCA+ patients at higher risk for chronic lung disease like pulmonary fibrosis or chronic respiratory symptoms? • Should cardiovascular disease risk be managed differently in patients who are MPO-ANCA+?

## Author Contributions

ZW and JS contributed to conception of the review, literature search, manuscript drafting and revision, read the review for publication, and agree to be accountable for all aspects of the review.

### Conflict of Interest

The authors declare that the research was conducted in the absence of any commercial or financial relationships that could be construed as a potential conflict of interest.
